# Naltrexone ameliorates functional network abnormalities in alcohol‐dependent individuals

**DOI:** 10.1111/adb.12503

**Published:** 2017-02-28

**Authors:** Laurel S. Morris, Kwangyeol Baek, Roger Tait, Rebecca Elliott, Karen D. Ersche, Remy Flechais, John McGonigle, Anna Murphy, Liam J. Nestor, Csaba Orban, Filippo Passetti, Louise M. Paterson, Ilan Rabiner, Laurence Reed, Dana Smith, John Suckling, Eleanor M. Taylor, Edward T. Bullmore, Anne R. Lingford‐Hughes, Bill Deakin, David J. Nutt, Barbara J. Sahakian, Trevor W. Robbins, Valerie Voon, ICCAM Consortium

**Affiliations:** ^1^ Department of Psychology University of Cambridge UK; ^2^ Behavioural and Clinical Neuroscience Institute University of Cambridge UK; ^3^ Department of Psychiatry University of Cambridge UK; ^4^ Centre for Neuropsychopharmacology, Division of Brain Sciences Imperial College London UK; ^5^ Neuroscience and Psychiatry Unit University of Manchester UK; ^6^ Imanova Centre for Imaging Sciences UK

**Keywords:** Addiction, alcohol, cocaine, naltrexone, opiate, substance use

## Abstract

Naltrexone, an opioid receptor antagonist, is commonly used as a relapse prevention medication in alcohol and opiate addiction, but its efficacy and the mechanisms underpinning its clinical usefulness are not well characterized. In the current study, we examined the effects of 50‐mg naltrexone compared with placebo on neural network changes associated with substance dependence in 21 alcohol and 36 poly‐drug‐dependent individuals compared with 36 healthy volunteers. Graph theoretic and network‐based statistical analysis of resting‐state functional magnetic resonance imaging (MRI) data revealed that alcohol‐dependent subjects had reduced functional connectivity of a dispersed network compared with both poly‐drug‐dependent and healthy subjects. Higher local efficiency was observed in both patient groups, indicating clustered and segregated network topology and information processing. Naltrexone normalized heightened local efficiency of the neural network in alcohol‐dependent individuals, to the same levels as healthy volunteers. Naltrexone failed to have an effect on the local efficiency in abstinent poly‐substance‐dependent individuals. Across groups, local efficiency was associated with substance, but no alcohol exposure implicating local efficiency as a potential premorbid risk factor in alcohol use disorders that can be ameliorated by naltrexone. These findings suggest one possible mechanism for the clinical effects of naltrexone, namely, the amelioration of disrupted network topology.

## Introduction

Substance use disorders are complex, multifaceted disorders often characterized by cycles of intoxication, withdrawal and craving, propagating substance‐seeking behaviours that persist despite often‐severe negative consequences. The social, health and economic costs of substance dependence (SD) are high (Nutt *et al.* 2010); yet the universal efficacy of currently available treatments is limited, and the mechanisms of action of such treatments are poorly understood (Sturgess *et al.* 2011; Tiffany *et al.* 2012). Naltrexone, an opioid receptor antagonist targeting particularly the mu‐opioid receptor (MOR) subtype (Verebey & Mule 1975; Schmidt *et al.* 1985), has been shown in randomized controlled trials to be effective in alcohol dependence (AD) but may be more effective in some individuals or subgroups and not others (Streeton & Whelan 2001) (Anton 2008; Sturgess *et al.* 2011). The current study aims to examine the effects of naltrexone on the neural network changes associated with SD, specifically in poly‐drug SD and AD.

Alcohol, like some other drugs of abuse, increases mesolimbic dopamine (DA) release in rodents and humans (Boileau *et al.* 2003), one mechanism by which it exerts its primary reinforcing effects (Dichiara & Imperato 1988; Everitt, Dickinson, & Robbins 2001). Activation and inhibition of the MOR in the ventral tegmental area regulates basal dopamine release in the nucleus accumbens (ventral striatum) (Spanagel, Herz, & Shippenberg 1992). MOR blockade in the ventral tegmental area inhibits dopamine release following alcohol intake and opioid antagonists reduce alcohol intake in rodents (Mitchell *et al.* 2009). Consistent with this preclinical literature, naltrexone has been shown to have a small‐to‐moderate effect size in reducing alcohol use in some (Garbutt *et al.* 1999; Bouza *et al.* 2004) but not all studies (Krystal *et al.* 2001). In heroin dependence, naltrexone blocks the physiological and psychological effects of heroin (Navaratnam *et al.* 1994; Brewer 2002; Brewer & Streel 2010), preventing relapse, particularly in the early detoxification phase (Foster, Brewer, & Steele 2003). Thus, while substantive literature implicates naltrexone as an effective treatment for opiate dependence, there are still gaps in our understanding of its efficacy and mechanisms in individuals with AD.

Although the focus for pharmacological interventions has been on the mesolimbic DA system (Dichiara & Imperato 1988; Wise 1988; Everitt *et al.* 2001), downstream neural adaptations following drug use and dependence also implicate a range of other cortical and subcortical regions in drug seeking (Volkow & Fowler 2000), suggesting subsequent larger scale network disorganization. Neural network organization can be examined based on synchronization of intrinsic fluctuations in neural activity during rest, referred to as functional connectivity. Indeed, aberrant resting‐state functional connectivity across the brain has been demonstrated in heroin (Liu *et al.* 2010; Yuan *et al.* 2010; Jiang *et al.* 2013b), cocaine (Gu *et al.* 2010; Kelly *et al.* 2011; Konova *et al.* 2013) and alcohol (Weiland *et al.* 2014) use disorders. Investigation of these functional properties of the brain may further our understanding of the mechanisms underlying the relapse prevention effects of naltrexone.

Properties of functional brain networks can be mathematically captured using graph theory analysis. This analysis method identifies and characterizes the topological organization of brain networks as a whole (Bullmore & Sporns 2009; He & Evans 2010), rather than based on a regional approach. Mathematical characteristics of the entire network can be obtained, which moves away from examining specific regions and considers the brain a single functioning network. In this paper, we focus on network efficiency, which is indicated by the two basic network properties, clustering coefficient and path length (Watts & Strogatz 1998; He & Evans 2010). A clustering coefficient describes the number of all neighbouring connections for each region in a network (node) as a function of the total possible number of connections. Thus, it represents a measure of the local efficiency of a network. Path length describes the average number of connections needed for any two nodes to link, so inversely indicating global efficiency. These two properties (local and global efficiency) can be used to categorize networks into regular, random or small world (He & Evans 2010), describing both specialized and diffuse information processing systems.

While limited, previous graph theory analyses of SD have revealed that heroin‐dependent individuals have distorted topological neural network properties in the form of more variable local interconnectivity (Yuan *et al.* 2010) and a shift to a more random network (Jiang *et al.* 2013b). A small sample of cocaine‐dependent individuals show reduced local efficiency (Wang *et al.* 2015). However, the opposite pattern has been demonstrated in nicotine dependence, of enhanced local efficiency and reduced global efficiency (Lin *et al.* 2015). Furthermore, a previous study reported no difference in local or global efficiency in heroin dependence (Jiang *et al.* 2013a). Thus, application of these methods to the study of abstinent AD and poly‐drug SD is limited, and the existing literature remains inconsistent.

In the current study, we examined the effect of naltrexone on global network properties, namely, local and global efficiency, assessing their relationship with addictive substance exposure. We also assessed region‐to‐region connectivity in a data‐driven manner with network‐based statistics (NBS) (Zalesky, Fornito, & Bullmore 2010) in individuals with AD, poly‐drug SD and healthy controls. If aberrant network organization is observed in AD and poly‐drug SD, restoration of such topological properties may be one mechanism by which naltrexone exerts its effects.

## Materials and Methods

### Participants

Details of study design and procedures are reported elsewhere (Paterson *et al.* 2015). Inclusion criteria included individuals who met DSM‐IV criteria for current or prior AD, or another substance of dependence (e.g. amphetamines, benzodiazepines, cocaine and opiates) (poly‐drug SD). Participants were abstinent, and there was no upper limit for abstinence. Abstinence was determined through clinical interviews during the baseline session and by using urine and alcohol breath test on each day of testing. Participants were not undergoing pharmacological treatment. All participants were aged 21 to 64 years. The healthy control group had no previous history of substance abuse, as assessed using the Alcohol, Smoking and Substance Involvement Screening Test (ASSIST) (Group WAW 2002) and timeline follow‐back. The healthy control group was matched for age, gender and smoking status where possible. All participants were required to provide a negative breath alcohol test and a negative urine sample (screening for the presence of amphetamines, benzodiazepines, cocaine and opiates) on experimental days. Participants were asked to refrain from cannabis use for the 7 days prior to testing. Positive results for cannabinoids were accepted; however, because of the long half‐life of metabolites, so long as the participant was not under intoxication or withdrawal. For the alcohol group, prior dependence on any other substance was exclusionary. Exclusion criteria included are as follows: current use of regular prescription or non‐prescription medication that would interfere with study integrity or subject safety; current primary axis I diagnosis; current or past history of enduring severe mental illness; current or past psychiatric history that contraindicated participation; history or current significant neurological diagnosis that may have influenced analysis or results; claustrophobia or unable to lie in the MRI scanner for 90 minutes; a cardiac pacemaker, other electronic device or other MRI contraindication, including pregnancy, as assessed by a standard pre‐MRI questionnaire. Secondary or lifetime history of depression or anxiety was permitted in both SD and healthy volunteers, as these are common comorbidities in the former.

### Procedure

All participants underwent a baseline session followed by two study sessions in which clinical, cognitive and neuroimaging tests were completed under either placebo or naltrexone, in counterbalanced order. The drug was administered 2 hours before each scan. A 50‐mg oral dose of naltrexone (manufactured by Bristol‐Myers Squibb Pharmaceutical Limited, UK) provided as per the British National Formulary was administered per participant. Subjects participated in resting state functional MRI, which was always first, and followed by three task‐based functional MRI scans. We examined individuals who completed both study sessions, which were counterbalanced, and there were no participant dropouts between these sessions (36 healthy volunteers [17 placebo first, Imperial/Cambridge/Manchester (ICM) = 13/12/11]; 36 poly‐drug SD (19 placebo first, ICM = 12/13/11); 21 AD (12 placebo first, ICM = 10/6/5)). Please see Supporting Information for further details of procedure and assessments taken.

### Resting State Functional MRI

#### Acquisition and Processing

Data acquisition procedures are reported elsewhere (McGonigle *et al.* 2016). Briefly, data were collected from three centres in the United Kingdom (Imperial College London and University of Cambridge with 3T Siemens Tim Trio with a Siemens 32 channel head coil, and Salford Royal NHS Foundation Trust, Manchester using 3T Philips Achieva with an eight‐element SENSE head coil). A recent report (McGonigle *et al.* 2016) demonstrated no differences between centres for neuroimaging results during task performance, using the exact same data acquisition sequences and processing in healthy volunteers. Please see Supporting Information for further details.

Resting state functional MRI data were collected for 360 seconds from all participants with eyes closed. Participants were asked to think of nothing in particular. Data were pre‐processed using speedypp.py as part of the fMRI signal processing toolbox v1.0, which draws upon modules from afni toolbox (http://afni.nimh.nih.gov). As motion significantly affects connectivity measures during resting state fMRI, motion parameters and derivatives and signal from cerebrospinal fluid were regressed out. Signals with high change in BOLD signal from volume to volume, i.e. dvars > 2, may not have been sufficiently corrected for motion (Power *et al.* 2012), and these subjects were removed in a secondary, more stringent analysis, resulting in removal of three AD, 10 poly‐drug SD and eight HV participants. Dvars were not significantly different between groups (*P* > 0.05). Data were zero‐padded, despiked and slice‐time corrected. Anatomical and functional images were co‐registered and normalized to a Montreal Neurological Institute (MNI152) template.

#### Graph Theory Analysis

We extracted regional mean fMRI time series for 90 regions of interest (ROI) in the Anatomical Automatic Labeling template (Tzourio‐Mazoyer *et al.* 2002) for each individual. Each regional mean time series was decomposed into wavelet coefficients at four scales using the maximum overlap discrete wavelet transform, a time‐frequency transformation. We used the scale 2 wavelet correlation matrices, which represent functionally relevant signals in the frequency range of 0.061~0.125 Hz, in line with previous reports assessing network efficiency measures (Achard & Bullmore 2007). We constructed a whole brain ROI‐to‐ROI correlation matrix using pairwise region‐to‐region correlations between coefficients, as described in a previous study (Achard & Bullmore 2007). The functional connectivity weights were binarized with the density threshold of 5 percent to control network density between subjects (Achard & Bullmore 2007) and reduce the possibility of spurious connections, thus being the most parsimonious threshold generating the core connectivity of the network.

#### Whole Brain Network Characteristics

Local and global efficiency in the binarized graph were computed as described in previous literature (Meunier *et al.* 2009; Rubinov & Sporns 2010). Local and global efficiency measures were entered into repeated measures ANOVA assessing group as a between‐subject factor and drug as a within‐subject factor. Comparisons were made using placebo, as a drug‐control session; however, the baseline session was additionally assessed to examine variability across sessions for each subject group. The AD and poly‐drug SD groups were then separately compared with the HV group. Local and global efficiency were correlated with drug and alcohol exposure using Pearson's correlation. Outcomes of *P* < 0.05 were considered significant. Nodal efficiency was examined on an exploratory basis for significant main findings.

#### Network‐based Statistics

Group comparisons in region‐to‐region connectivity were assessed using NBS Toolbox (Zalesky *et al.* 2010), for MATLAB (The MathWorks, Inc., Natick, Massachusetts, United States). Group differences are tested at every connection within the connectivity matrices (inter‐regional correlation in BOLD activity) using an initial threshold of *T* > 3 as described in the work of Zalesky *et al* (2010). NBS identified the interconnected subnetwork consisting of suprathreshold edges (*T* > 3 in group difference). The size of the interconnected subnetwork (i.e. the number of interconnected suprathreshold edges) was used to calculate a family‐wise error corrected *P*‐value using 10 000 permutation tests (*P* < 0.05, family‐wise error corrected). In other words, NBS identified an interconnected subnetwork of altered connectivity with a cluster‐level corrected *P*‐values using network cluster size (the number of interconnected suprathreshold edges). AD and poly‐drug SD were separately compared with HV. AD and poly‐drug SD were compared with each other on an exploratory basis.

## Results

### Participant Characteristics

Demographic and questionnaire data are presented in Table 1. Of the poly‐drug SD, 12 met criteria for all of alcohol, cocaine and opiate dependence, eight for alcohol and cocaine, three for alcohol and opiates and five for cocaine or opiates only. Poly‐drug SD were 31.493 ± 33.750 months abstinent from cocaine (range 0.5–132) and 19.833 ± 4.326 age of first use; were 18.157 ± 37.483 months abstinent from alcohol (range: 0.5–204); and were 42.683 ± 59.684 months abstinent from opiates (range: 1.5–276) and age of first opiate use was 20.767 ± 6.038. Healthy volunteers and AD had no history of opiate use. Five healthy volunteers and 10 AD had a history of cocaine use without meeting criteria for dependence. AD participants were abstinent from alcohol 13.417 ± 18.693 months (range: 0.5–79), and age of regular alcohol use was 16.524 ± 3.558, and abstinent from cocaine 28.955 ± 24.942 months (range: 1.75–72) and age of first use of cocaine was 31.455 ± 10.182. Healthy volunteers were 0.317 ± 0.924 months abstinent from alcohol (range: 0–0.75) and 120.6 ± 140.225 from cocaine (range: 3–360). Further details including smoking information are presented in Table 1.

**Table 1:** Demographic and questionnaire data.

	*n*	Age	Sex (female)	BDI‐II‐total	STAI‐S‐total	STAI‐T‐total
Poly‐drug SD	36	38.167	7.000	10.889	34.194	40.972
		7.636		6.927	9.674	12.263
AD	21	45.381	4.000	9.905	33.333	40.905
		8.789		9.534	11.284	12.136
HV	36	40.833	8.000	3.861	26.472	29.944
		8.674		4.486	6.759	7.917
*P*		0.011		<0.001	0.001	<0.001
	Alcohol abstinence	Cocaine abstinence	Opiate abstinence	Alcohol exposure	Cocaine exposure	Opiate exposure
Poly‐drug SD	18.157	31.493	42.683	9.903	6.153	7.867
	37.483	33.750	59.684	9.260	5.399	7.234
AD	13.417	28.955	15.875	18.714	0.636	0
	18.693	24.942	19.976	8.655	1.433	0
HV	0.317	120.600	NA	1.176	0	0
	0.924	140.335	NA	3.157	0	0
	Age first used cocaine	Age first used opiates	Age used alcohol regularly	Age used cocaine regularly	Age used opiates regularly	
Poly‐drug SD	19.833	20.767	15.559	23.594	21.750	
	4.326	6.038	3.910	6.293	3.904	
AD	31.455	42.000	16.524	33.000		
	10.182	7.071	3.558	9.592		
HV	25.000	NA	18.485	Na	NA	
	2.739	NA	3.684	NA	NA	
	Smokers (current/previous/non)	Cigarettes number per day	Pack years			
Poly‐drug SD	08/04/24	15.964	19.541			
		8.558	14.261			
AD	14/04/03	17.179	26.535			
		7.324	18.404			
HV	19/04/13	9.637	14.667			
		6.776	12.623			
*p*	0.004	0.010	0.052			

Mean and standard deviation is reported. Abstinence is reported in months.

SD, substance dependence; AD, alcohol dependence; HV, healthy volunteer; N, number; BDI, Beck Depression Inventory; STAI, state–trait anxiety index; NA, not applicable.

### Whole Brain Network Characteristics

We examined local and global efficiency of neural networks in AD, poly‐drug SD and healthy volunteers (HV) comparing between naltrexone and placebo as a drug control. There was a significant effect of drug (F_(1,90)_ = 7.977, *P* = 0.006), and a significant drug × group interaction (F_(2,90)_ = 3.672, *P* = 0.029). There was no effect of group (F_(1,90)_ = 2.306, *P* = 0.106). Results remained significant when 21 participants were removed based on a stringent motion threshold (without 3 AD, 10 poly‐drug SD and 8 HV: effect of naltrexone, *P* = 0.006; interaction, *P* = 0.041). Results remained significant when HV with any prior drug use were removed from the analysis and when opiate or cocaine dependence only was removed (i.e. ‘pure’ HV and poly‐drug dependence). Analysis of global efficiency showed no group, naltrexone effects or interactions.

**Figure 1:**
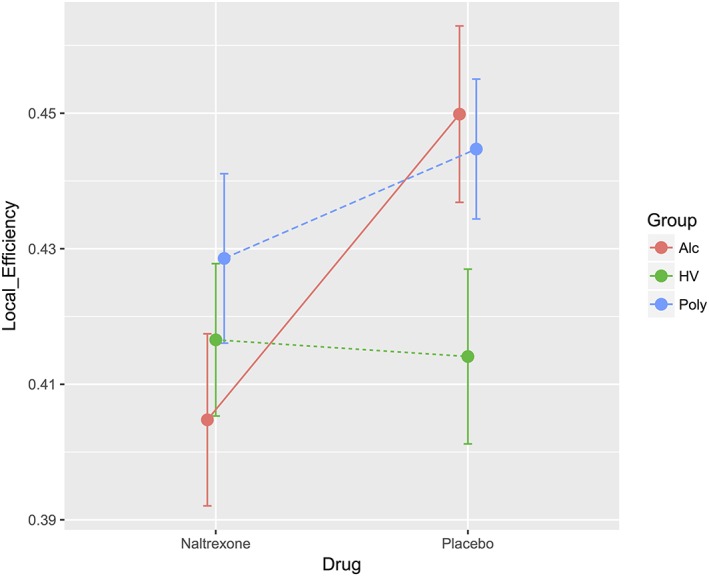
Neural network local efficiency under placebo and naltrexone. Local efficiency was captured based on a whole brain ROI‐to‐ROI correlation coefficient matrix, binarized with a 5 percent density threshold and is plotted for naltrexone and placebo for alcohol‐dependent (Alc, unbroken line), poly‐substance‐dependent (Poly, broken line) and healthy volunteers (HV, dotted line). For the comparison between drugs, there was a significant effect of naltrexone (*P* = 0.006), a significant drug × group interaction (*P* = 0.029) and no effect of group (*P* = 0.106)

For the investigation of the effects on local efficiency further, the whole AD and poly‐drug SD were separately compared with the whole HV group. For the AD comparison with healthy volunteers, there was a main effect of drug (F_(1,55)_ = 5.610, *P* = 0.021) in which naltrexone was associated with lower local efficiency. There was also a significant group × drug interaction (F_(1,55)_ = 6.836, *P* = 0.012) (Fig. 1) in which naltrexone decreased local efficiency to a greater extent in AD, bringing local efficiency to the same level as healthy volunteers. For the examination of which regions contributed to this difference in local efficiency, nodal efficiency for individual nodes was compared on an exploratory basis. AD had increased local efficiency of bilateral rectus gyrus (medial orbitofrontal cortex), right supplementary motor area, left middle frontal gyrus, left middle occipital gyrus, left parahippocampal gyrus and right olfactory bulb nodes during placebo and increased local efficiency in the middle cingulate gyrus only during naltrexone; however, these findings did not survive Bonferroni correction for multiple comparisons (Supporting Information Table S1). The main effects were also replicated in an analysis with 21 participants removed based on stringent motion thresholds (naltrexone effect, *P* = 0.034; interaction, *P* = 0.015). There was no main effect of group (F_(1,55)_ = 1.399, *P* = 0.242).

For the poly‐drug SD comparison with healthy volunteers, there was a main effect of group (F_(1,70)_ = 4.259, *P* = 0.043) in which poly‐drug SD was associated with higher local efficiency, suggesting more segregated and clustered information processing. There was no main effect of naltrexone (F_(1,70)_ = 0.845, *P* = 0.361) or interaction (F_(1,70)_ = 1.563, *P* = 0.215). Removing 10 poly‐drug SD and eight HV individuals from this analysis based on stringent motion criteria resulted in a lack of significant group effect (*P* = 0.159).

Baseline local efficiency was also computed and illustrated in Supporting Information Fig. S1. Across the three sessions (baseline, placebo and naltrexone), there was no difference in local efficiency in HV (F_(2,33)_ = 0.486, *P* = 0.619) or poly‐drug SD subjects (F_(2,33)_ = 2.182, *P* = 0.129). However, there was a significant difference across sessions in AD (F_(2,17)_ = 5.439, *P* = 0.015, Supporting Information Fig. S1), in which naltrexone reduced or ‘normalized’ local efficiency in this group only.

### Clinical Relationship

**Figure 2:**
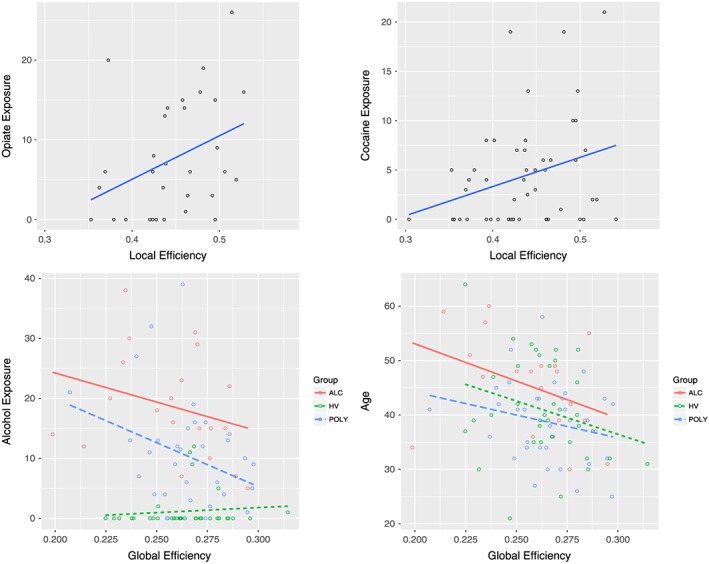
Correlations between neural network efficiency and drug exposure. Top: local efficiency is plotted against opiate exposure (*R* = 0.365, *P* = 0.044) and cocaine exposure (*R* = 0.295, *P* = 0.058). Bottom: global efficiency plotted against alcohol exposure and age

We further examined the relationship between drug/addictive substance exposure and local and global efficiency on placebo. Local efficiency positively correlated with opiate exposure (*R* = 0.365, *P* = 0.044) and cocaine exposure at trend level (*R* = 0.295, *P* = 0.058) across AD and poly‐drug SD groups (Fig. 2). While there were no group differences in global efficiency, we found that across all groups including healthy volunteers it negatively correlated with alcohol exposure (*R* = −0.210, *P* = 0.049) and age (*R* = −0.292, *P* = 0.005) (Fig. 2). When controlling for age, there was no significant correlation between global efficiency and alcohol exposure, suggesting that age might have been the contributing factor to reduced global efficiency as the AD group was slightly older.

Local efficiency was not correlated with age, length of abstinence from alcohol across AD and poly‐drug SD groups (*P* > 0.6) or from cocaine (*P* > 0.1) or opiates (*P* > 0.8) in the poly‐drug SD group. As levels of depressive and anxiety symptoms were higher in AD and poly‐drug SD groups (Table 1), we examined whether there was any relationship between these measures and network efficiency. BDI‐II scores for depression and state and trait STAI anxiety scores were not significantly correlated with local or global efficiency on placebo or naltrexone (*P* > 0.1). Please see Supplementary materials for further data.

### Network‐based Statistics

**Figure 3:**
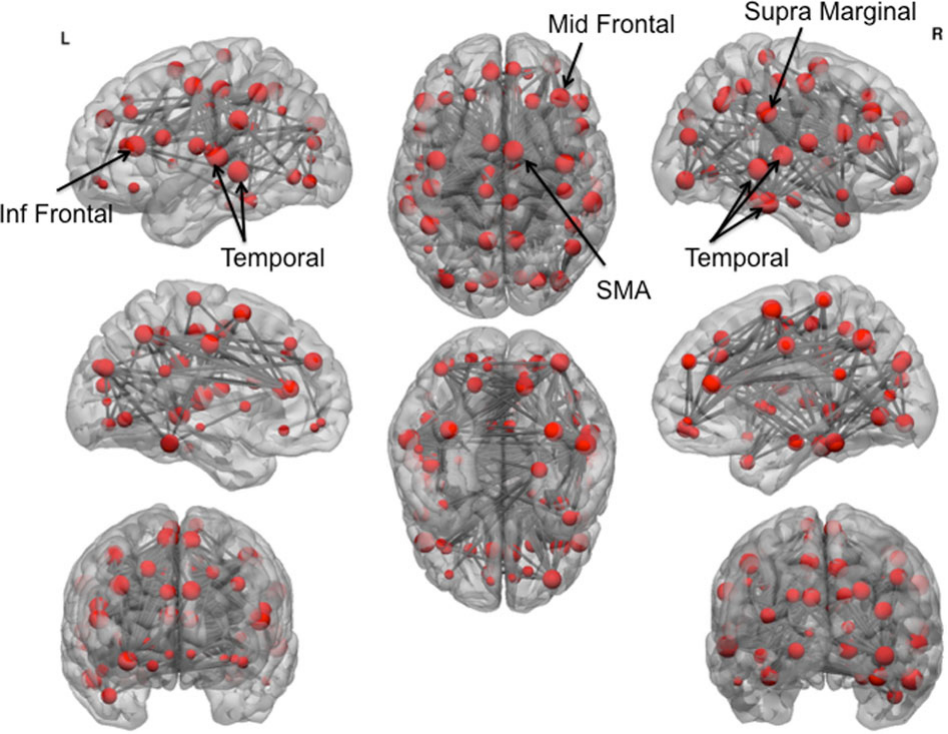
Network cluster of reduced functional connectivity in alcohol‐dependent subjects. Network‐based statistics demonstrated a large network of reduced connectivity in alcohol‐dependent compared with healthy subjects. Node size indicates number of connections with reduced functional connectivity. The largest nodes are annotated. Inf, inferior; SMA, supplementary motor area; L, left; R, right

**Figure 4:**
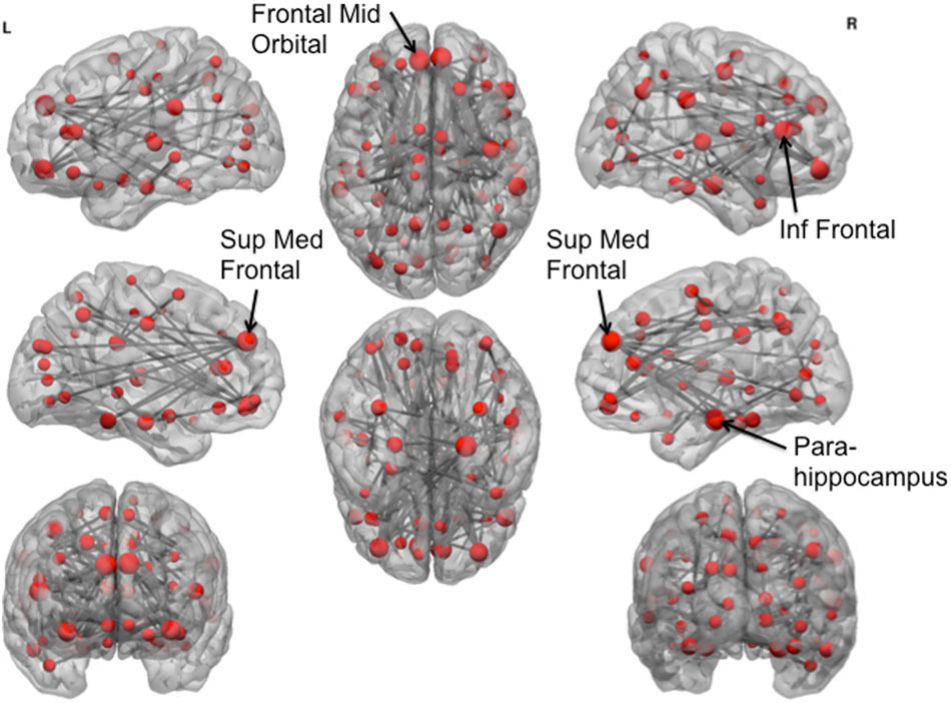
Network cluster of reduced functional connectivity in alcohol‐dependent subjects compared with poly‐drug substance‐dependent subjects. Network‐based statistics demonstrated a network of reduced connectivity in alcohol‐dependent subjects compared with poly‐drug SD subjects. Node size indicates number of connections with reduced functional connectivity. The largest nodes are annotated. Sup, superior; Med, medial; Inf, inferior; L, left; R, right

In order to understand which regions might be driving this disruption in local efficiency, we examined the subnetwork of altered functional connectivity using network‐based statistical analysis. AD and poly‐drug SD were separately compared with HV. NBS identified a large network of significantly decreased functional connectivity in AD compared with HV (*P* = 0.001, network‐based, 69 nodes, 373 edges) (Fig. 3, Supporting Information Table S2) during placebo. The network cluster included many regions, but regions showing the greatest reduction in connectivity were temporal, inferior frontal and supplementary motor area nodes (depicted as larger nodes in Fig. 3). There was no significantly increased functional connectivity in AD compared with HV with NBS. There was no significant difference between the poly‐drug SD group and HV as measured by NBS (*P* > 0.1). The reduction in network connectivity in AD compared with HV was replicated in the baseline session (*P* = 0.030, 40 nodes, 53 edges, Supporting Information Table S4 and Fig. S2), although the network cluster included different regions. The lack of difference between poly‐drug SD and HV was also replicated at baseline (*P* > 0.05). On an exploratory basis, we also directly compared between the poly‐drug SD and AD groups. AD had reduced network connectivity compared with poly‐drug SD in a network that comprised of frontal regions and para‐hippocampus (*P* = 0.010, 61 nodes, 111 edges, Fig. 4 and Supporting Information Table S3).

## Discussion

We examined the effects of a single dose of the MOR antagonist naltrexone in AD and poly‐drug SD subjects. AD had elevated local efficiency across the whole neural network indicating more isolated and clustered information processing. Naltrexone decreased local efficiency in AD individuals, to the same levels as healthy volunteers. Local efficiency in poly‐drug SD was not affected by naltrexone and was positively associated with exposure to drugs of abuse (opiate exposure and a trend for cocaine exposure). That naltrexone normalizes local efficiency in AD but no poly‐drug SD may help determine likely respondents to pharmacotherapy and the current imaging techniques may highlight potential responders.

Naltrexone seemed to be effective at reducing local efficiency in the AD group but had no effect in poly‐drug SD subjects. Indeed, poly‐drug SD subjects showed no difference in local efficiency across the three sessions, although they did show heightened local efficiency during placebo. Interpretation of these findings is limited by the comorbidity within this group, and we caution that further investigation of local efficiency in poly‐drug SD is necessary. For example, heroin‐dependent individuals show a shift to a more random network (Jiang *et al.* 2013b), but cocaine‐dependent individuals show reduced local efficiency (Wang *et al.* 2015). However, the current study does clarify the effect of naltrexone on local efficiency in AD subjects. While healthy individuals demonstrated stable levels of local efficiency throughout the experiment, AD showed heighted local efficiency during baseline and placebo that was significantly reduced under naltrexone.

The higher local efficiency observed suggests elevated clustering of functionally related regions (Sporns *et al.* 2004). This means that information processing within certain neural networks is stronger, with less cross‐talk between distinct functional processes. This clustering of connections suggests hyperactive intermediate signalling (rather than long‐range signalling) in the network, perturbing the capacity for more global neural cooperation or wider cortical interactions (Sporns *et al.* 2004). Higher local efficiency may also suggest higher likelihood of closed feedback loops and enhanced reciprocity of connections, meaning that information is not dispersed and processed across networks but remains closed off in a specific network model (Sporns *et al.* 2004). Thus, information process is more segregated and clustered. Higher segregation of network communication may relate to less flexible information processing, a functional segregation and behavioural rigidity that is observed in addiction. The medial orbitofrontal cortex and supplementary motor area were particularly implicated as being more segregated. Indeed, AD individuals have reduced volume and cortical thickness of the orbitofrontal cortex (Durazzo *et al.* 2011) that predicts future relapse (Beck *et al.* 2012) and simultaneously show heightened drug cue reactivity in this region (Chase *et al.* 2011; Kuhn & Gallinat 2011; Engelmann *et al.* 2012), which is modulated by gene type during naltrexone (OPRM1 gene G allele carriers) (Kareken *et al.* 2010). Similarly, supplementary motor area volume is reduced in binge drinkers (Kvamme *et al.* 2016), and AD subjects show increased SMA activity related to impulsivity (Claus, Kiehl, & Hutchison 2011). The current demonstration of network topology changes in these regions may reflect reduced long‐range connections but more local neighbouring connections that coincide with reduced volumes but increased functional responses to drug or salient cues.

While global network measures like local efficiency, and region‐to‐region connectivity measures are quite distinct, one might be used theoretically to inform the other. In the current paper, NBS were used to examine which nodes of the network might be driving the finding of heightened local efficiency in the AD group. AD demonstrated a significant reduction in connectivity in a network involving frontal and temporal and supplementary motor regions, compared with HV. This might suggest that frontal and temporal regions show enhanced clustering and segregation of information processing that would simultaneously contribute to reduced functional connectivity and higher global local efficiency. Furthermore, the decreased functional connectivity in AD revealed by NBS may be long‐range, cross‐module connections. A reduction in long‐range connections is associated with more isolated local circuits, making circuit modules highly clustered, thereby increasing local efficiency. Reduced neural functional connectivity has been previously demonstrated in this group, particularly of the left executive control network (including parietal, dorsolateral and medial prefrontal cortex), i.e. associated with impaired behavioural control and alcohol use severity (Weiland *et al.* 2014). Furthermore, in young adults at high risk for AD, enhanced task‐related functional connectivity among nucleus accumbens, sensorimotor cortex and precuneus has been associated with alcohol use severity (Weiland *et al.* 2014). These findings suggest a reduced baseline state and hyperactive task‐related connectivity of particular nodes may relate to more severe alcohol use.

There have been several characterizations of the effects of stimulant and opiate SD on seed‐based functional connectivity. Chronic heroin users show disruption of both cortical (prefrontal cortex, anterior cingulate and insula) and subcortical (ventral striatum, amygdala and hippocampus) connectivity patterns (Liu *et al.* 2010; Yuan *et al.* 2010; Jiang *et al.* 2013b), consistent with deficits in executive decision making (Verdejo‐Garcia, Perales, & Perez‐Garcia 2007; Verdejo‐Garcia & Perez‐Garcia 2007) and reward‐related behavioural control (Harris & Aston‐Jones 2003) in this group. Similarly, cocaine‐dependent individuals have reduced functional connectivity of widespread prefrontal, premotor and parietal networks associated with attentional deficits (see also (Gu *et al.* 2010; Konova *et al.* 2013)). However, few studies have focused on poly‐drug SD, and as mentioned, comorbidity might be an issue because of distinct effects of each substance of abuse on topological or functional network properties. The current findings do suggest that prolonged drug exposure in the poly‐drug SD group, in particular exposure to opiates, is associated with elevated local efficiency of neural networks. That local efficiency is related to opiate and cocaine use, but no alcohol suggests local efficiency as a potential premorbid risk factor in AD that can be ameliorated by naltrexone whereas naltrexone does not modulate local efficiency in poly‐drug SD.

In contrast with a previous study of local and global efficiency in cocaine‐dependent individuals (Wang *et al.* 2015), we did not find group differences in global efficiency. Inconsistencies between the findings of the current and previous studies may relate to the presentation of poly‐drug use rather than single‐drug dependence, or relate to abstinence. The previous study showing reduced global efficiency in cocaine‐dependent individuals included individuals with 4 to 8 days of abstinence rather than the current criteria of at least 4 weeks. Further studies are clearly required to elucidate the effects of substance and abstinence on large‐scale network efficiency measures.

Limitations of the current study include the comorbidity within groups, e.g. nicotine use or dependence and high levels of depression and anxiety. This however is a fair representation of the substance and alcohol‐dependent population as a whole. While the current study uses data from three separate centres, this can be considered a benefit rather than a limitation as it demonstrates, along with a previous report (McGonigle *et al.* 2016), that data can be integrated effectively across sites, a feature that is particularly important for large studies examining groups with high attrition rates.

We have thus provided insight into the effects (or lack thereof) of a well‐established psychopharmaceutical agent, naltrexone, on large‐scale network dynamics in AD and poly‐drug SD subjects. Differentiating drug efficacy profiles for distinct groups of patients is crucial for the development of more effective and targeted treatments.

## Funding and Disclosure

The research was supported by the NIHR CRF at Imperial College Healthcare NHS Trust, the NIHR/Wellcome Trust Cambridge Research Facility and Clinical Trials Unit at Salford Royal NHS Foundation Trust. The study is supported by the North West London, Eastern and Greater Manchester NIHR Clinical Research Networks. The views expressed are those of the author(s) and not necessarily those of the Medical Research Council, the NHS, the NIHR or the Department of Health.

The authors declared the following potential conflicts of interest regarding the research, authorship and/or publication of this article. David Nutt is an advisor to British National Formulary, MRC, General Medical Council, Department of Health; is President of the European Brain Council; is past President of the British Neuroscience Association and European College of Neuropsychopharmacology; is chair of the Independent Scientific Committee on Drugs (UK); is a member of the International Centre for Science in Drug Policy; is advisor to Swedish government on drug, alcohol and tobacco research, editor of the Journal of Psychopharmacology; sits on advisory Boards at Lundbeck, MSD, Nalpharm, Orexigen, Shire; has received speaking honoraria (in addition to previously) from BMS/Otsuka, GSK, Lilly, Janssen, Servier; is a member of the Lundbeck International Neuroscience Foundation; has received grants or clinical trial payments from P1vital, MRC, NHS, Lundbeck; has share options with P1vital; has been expert witness in a number of legal cases relating to psychotropic drugs; and has edited/written 27 books, some purchased by pharmaceutical companies. Trevor W Robbins has research grants with Eli Lilly and Lundbeck; has received royalties from Cambridge Cognition (CANTAB); has received editorial honoraria from Springer Verlag, Elsevier, Society for Neuroscience; has performed educational lectures for Merck, Sharpe and Dohme; and does consultancy work for Cambridge Cognition, Eli Lilly, Lundbeck, Teva and Shire Pharmaceuticals.

JF William Deakin currently advises or carries out research funded by Autifony, Sunovion, Lundbeck, AstraZeneca and Servier. All payment is to the University of Manchester. Anne Lingford‐Hughes has received honoraria from Lundbeck and research support from GSK for a PhD studentship. Liam Nestor was employed by GSK during some of this work. John Suckling has received research support from GSK. Barbara J Sahakian consults for Cambridge Cognition, Peak (Brainbow), and Servier, Otsuka and Lundbeck. She holds a grant from Janssen/J&J. She holds shares in CeNeS and share options in Cambridge Cognition. The remaining authors declare no potential conflicts of interest.

The following financial support was received for the research, authorship and/or publication of this article: independent research funded by the MRC as part of the addiction initiative (grant number G1000018); GSK funded the functional and structural MRI scans at Imperial College.

## Authors Contribution

ALH, DJN, BS, TWR, RE, BD, LSM and VV were responsible for the study concept. IR, JS, DS, LP, RF, ET, LR, CO, JM, AM LJN and RT contributed to the study design and acquisition of the data. LSM, KB and RT analysed the data. JS, VV and KDE assisted with the analysis of the data. LSM, VV and TWR assisted the interpretation of the findings. LSM drafted the manuscript. VV and TWR provided critical revision of the manuscript. All authors critically reviewed the content and approved the final version for publication.

## Supporting information


**Table S1.** Regions with reduced nodal efficiency in alcohol dependent (Alc) individuals compared to healthy volunteers (HV). Local efficiency for each node was calculated and compared between groups during placebo and naltrexone. Mean and standard error of the mean (s.e.m) are demonstrated and *P*‐values for both uncorrected and Bonferroni correction for multiple comparisons tests are displayed. Left, L; Right, R. Table S2. Regions demonstrating reduced functional connectivity in alcohol dependent subjects compared to healthy volunteers. Network based statistics revealed a significant cluster of reduced functional connectivity including 69 nodes and 373 edges. The number indicates the number of connections included in the significantly reduced cluster. Temporal and inferior frontal regions seemed to show the highest reduction in network connectivity. Left, L; Right, R. Table S3. Regions demonstrating reduced functional connectivity in alcohol dependent subjects compared to poly‐drug dependent subjects. The number indicates the number of connections included in the significantly reduced cluster. Frontal regions show the highest reduction in network connectivity. Left, L; Right, R. Table S4. Regions demonstrating reduced functional connectivity in alcohol dependent subjects compared to healthy volunteers during baseline session. The number indicates the number of connections included in the significantly reduced cluster. Left, L; Right, R. Table S5. Regions demonstrating reduced functional connectivity in alcohol dependent subjects compared to healthy volunteers during naltrexone. The number indicates the number of connections included in the significantly reduced cluster. Left, L; Right, R

**Figure S1.** Neural network local efficiency under naltrexone. Local efficiency was captured based on a whole brain ROI‐to‐ROI correlation coefficient matrix, binarized with a 5 percent density threshold and is plotted for baseline, naltrexone and placebo for alcohol dependent (Alc, unbroken line), poly‐substance dependent (Poly, broken line) and healthy volunteers (HV, dotted line). There was no difference in local efficiency across sessions in HV or Polysubjects. There was a significant difference across sessions in the Alc group, in which local efficiency was significantly reduced by naltrexone

**Figure S2.** Network cluster of reduced functional connectivity in alcohol dependent (AD) subjects during baseline session. Network based statistics demonstrated a large network of reduced connectivity in AD compared with healthy subjects. Node size indicates number of connections with reduced functional connectivity. The largest nodes are annotated

## References

[adb12503-bib-0001] Achard S , Bullmore E (2007) Efficiency and cost of economical brain functional networks. PLoS Comput Biol 3:e17. 17274684 10.1371/journal.pcbi.0030017 PMC1794324

[adb12503-bib-0002] Anton RF (2008) Naltrexone for the management of alcohol dependence. New England J Med 359:715–721. 18703474 10.1056/NEJMct0801733 PMC2565602

[adb12503-bib-0003] Beck A , Wustenberg T , Genauck A , Wrase J , Schlagenhauf F , Smolka MN , Mann K , Heinz A (2012) Effect of brain structure, brain function, and brain connectivity on relapse in alcohol‐dependent patients. Arch Gen Psychiatry 69:842–852. 22868938 10.1001/archgenpsychiatry.2011.2026

[adb12503-bib-0004] Boileau I , Assaad JM , Pihl RO , Benkelfat C , Leyton M , Diksic M , Tremblay RE , Dagher A (2003) Alcohol promotes dopamine release in the human nucleus accumbens. Synapse 49:226–231. 12827641 10.1002/syn.10226

[adb12503-bib-0005] Bouza C , Angeles M , Munoz A , Amate JM (2004) Efficacy and safety of naltrexone and acamprosate in the treatment of alcohol dependence: a systematic review. Addiction 99:811–828. 15200577 10.1111/j.1360-0443.2004.00763.x

[adb12503-bib-0006] Brewer C (2002) Serum naltrexone and 6‐beta‐naltrexol levels from naltrexone implants can block very large amounts of heroin: a report of two cases. Addict Biol 7:321–323. 12126492 10.1080/13556210220139541

[adb12503-bib-0007] Brewer C , Streel E (2010) Recent developments in naltrexone implants and depot injections for opiate abuse: the new kid on the block is approaching adulthood. Adicciones 22:285–291. 21152846

[adb12503-bib-0008] Bullmore E , Sporns O (2009) Complex brain networks: graph theoretical analysis of structural and functional systems (vol 10, pg 186, 2009). Nat Rev Neurosci 10. 10.1038/nrn2575 19190637

[adb12503-bib-0009] Chase HW , Eickhoff SB , Laird AR , Hogarth L (2011) The neural basis of drug stimulus processing and craving: an activation likelihood estimation meta‐analysis. Biol Psychiatry 70:785–793. 21757184 10.1016/j.biopsych.2011.05.025 PMC4827617

[adb12503-bib-0010] Claus ED , Kiehl KA , Hutchison KE (2011) Neural and behavioral mechanisms of impulsive choice in alcohol use disorder. Alcohol Clin Exp Res 35:1209–1219. 21676001 10.1111/j.1530-0277.2011.01455.x PMC3117198

[adb12503-bib-0011] Dichiara G , Imperato A (1988) Drugs abused by humans preferentially increase synaptic dopamine concentrations in the mesolimbic system of freely moving rats. Proc Natl Acad Sci U S A 85:5274–5278. 2899326 10.1073/pnas.85.14.5274 PMC281732

[adb12503-bib-0012] Durazzo TC , Tosun D , Buckley S , Gazdzinski S , Mon A , Fryer SL , Meyerhoff DJ (2011) Cortical thickness, surface area, and volume of the brain reward system in alcohol dependence: relationships to relapse and extended abstinence. Alcohol Clin Exp Res 35:1187–1200. 21410483 10.1111/j.1530-0277.2011.01452.x PMC3097308

[adb12503-bib-0013] Engelmann JM , Versace F , Robinson JD , Minnix JA , Lam CY , Cui Y , Brown VL , Cinciripini PM (2012) Neural substrates of smoking cue reactivity: a meta‐analysis of fMRI studies. Neuroimage 60:252–262. 22206965 10.1016/j.neuroimage.2011.12.024 PMC3288122

[adb12503-bib-0014] Everitt BJ , Dickinson A , Robbins TW (2001) The neuropsychological basis of addictive behaviour. Brain Res Rev 36:129–138. 11690609 10.1016/s0165-0173(01)00088-1

[adb12503-bib-0015] Foster J , Brewer C , Steele T (2003) Naltrexone implants can completely prevent early (1‐month) relapse after opiate detoxification: a pilot study of two cohorts totalling 101 patients with a note on naltrexone blood levels. Addict Biol 8:211–217. 12850780 10.1080/1355621031000117446

[adb12503-bib-0016] Garbutt JC , West SL , Carey TS , Lohr KN , Crews FT (1999) Pharmacological treatment of alcohol dependence: a review of the evidence. JAMA 281:1318–1325. 10208148 10.1001/jama.281.14.1318

[adb12503-bib-0017] Group WAW (2002) The alcohol, smoking and substance involvement screening test (ASSIST): development, reliability and feasibility. Addiction 97:1183–1194. 12199834 10.1046/j.1360-0443.2002.00185.x

[adb12503-bib-0018] Gu H , Salmeron BJ , Ross TJ , Geng XJ , Zhan W , Stein EA , Yang YH (2010) Mesocorticolimbic circuits are impaired in chronic cocaine users as demonstrated by resting‐state functional connectivity. Neuroimage 53:593–601. 20603217 10.1016/j.neuroimage.2010.06.066 PMC2930044

[adb12503-bib-0019] Harris GC , Aston‐Jones G (2003) Altered motivation and learning following opiate withdrawal: evidence for prolonged dysregulation of reward processing. Neuropsychopharmacology: official publication of the American College of Neuropsychopharmacology 28:865–871. 12736632 10.1038/sj.npp.1300122

[adb12503-bib-0020] He Y , Evans A (2010) Graph theoretical modeling of brain connectivity. Curr Opin Neurol 23:341–350. 20581686 10.1097/WCO.0b013e32833aa567

[adb12503-bib-0021] Jiang G , Wen X , Qiu Y , Zhang R , Wang J , Li M , Ma X , Tian J , Huang R (2013a) Disrupted topological organization in whole‐brain functional networks of heroin‐dependent individuals: a resting‐state fMRI study. PLoS One 8:e82715. 24358220 10.1371/journal.pone.0082715 PMC3866189

[adb12503-bib-0022] Jiang GH , Wen X , Qiu YW , Zhang RB , Wang JJ , Li M , Ma XF , Tian JZ , Huang RW (2013b) Disrupted topological organization in whole‐brain functional networks of heroin‐dependent individuals: a resting‐state fMRI study. PLoS One 8:e82715. 24358220 10.1371/journal.pone.0082715 PMC3866189

[adb12503-bib-0023] Kareken DA , Liang T , Wetherill L , Dzemidzic M , Bragulat V , Cox C , Talavage T , O'Connor SJ , Foroud T (2010) A polymorphism in GABRA2 is associated with the medial frontal response to alcohol cues in an fMRI study. Alcohol Clin Exp Res 34:2169–2178. 20698837 10.1111/j.1530-0277.2010.01293.x PMC4154567

[adb12503-bib-0024] Kelly C , Zuo XN , Gotimer K , Cox CL , Lynch L , Brock D , Imperati D , Garavan H , Rotrosen J , Castellanos FX , Milham MP (2011) Reduced interhemispheric resting state functional connectivity in cocaine addiction. Biol Psychiatry 69:684–692. 21251646 10.1016/j.biopsych.2010.11.022 PMC3056937

[adb12503-bib-0025] Konova AB , Moeller SJ , Tomasi D , Volkow ND , Goldstein RZ (2013) Effects of methylphenidate on resting‐state functional connectivity of the mesocorticolimbic dopamine pathways in cocaine addiction. JAMA Psychiat 70:857–868. 10.1001/jamapsychiatry.2013.1129 PMC4358734 23803700

[adb12503-bib-0026] Krystal JH , Cramer JA , Krol WF , Kirk GF , Rosenheck RA , Veterans Affairs Naltrexone Cooperative Study G (2001) Naltrexone in the treatment of alcohol dependence. N Engl J Med 345:1734–1739. 11742047 10.1056/NEJMoa011127

[adb12503-bib-0027] Kuhn S , Gallinat J (2011) Common biology of craving across legal and illegal drugs ‐ a quantitative meta‐analysis of cue‐reactivity brain response. Eur J Neurosci 33:1318–1326. 21261758 10.1111/j.1460-9568.2010.07590.x

[adb12503-bib-0028] Kvamme TL , Schmidt C , Strelchuk D , Chang‐Webb YC , Baek K , Voon V (2016) Sexually dimorphic brain volume interaction in college‐aged binge drinkers. NeuroImage Clinical 10:310–317. 26900571 10.1016/j.nicl.2015.12.004 PMC4724035

[adb12503-bib-0029] Lin FC , Wu GY , Zhu L , Lei H (2015) Altered brain functional networks in heavy smokers. Addict Biol 20:809–819. 24962385 10.1111/adb.12155

[adb12503-bib-0030] Liu J , Gao XP , Osunde I , Li X , Zhou SK , Zheng HR , Li LJ (2010) Increased regional homogeneity in internet addiction disorder: a resting state functional magnetic resonance imaging study. Chin Med J‐Peking 123:1904–1908. 20819576

[adb12503-bib-0031] McGonigle J , Murphy A , Paterson LM , Reed LJ , Nestor L , Nash J , Elliott R , Ersche KD , Flechais RS , Newbould R , Orban C , Smith DG , Taylor EM , Waldman AD , Robbins TW , Deakin JW , Nutt DJ , Lingford‐Hughes AR , Suckling J , Platform I (2016) The ICCAM platform study: an experimental medicine platform for evaluating new drugs for relapse prevention in addiction. Part B: fMRI description. J Psychopharmacol. 10.1177/0269881116668592 PMC5367542 27703042

[adb12503-bib-0032] Meunier D , Lambiotte R , Fornito A , Ersche KD , Bullmore ET (2009) Hierarchical modularity in human brain functional networks. Front Neuroinform 3:15–26. 19949480 10.3389/neuro.11.037.2009 PMC2784301

[adb12503-bib-0033] Mitchell JM , Bergren LJ , Chen KS , Rowbotham MC , Fields HL (2009) Naltrexone aversion and treatment efficacy are greatest in humans and rats that actively consume high levels of alcohol. Neurobiol Dis 33:72–80. 18955144 10.1016/j.nbd.2008.09.018

[adb12503-bib-0034] Navaratnam V , Jamaludin A , Raman N , Mohamed M , Mansor SM (1994) Determination of naltrexone dosage for narcotic agonist blockade in detoxified Asian addicts. Drug Alcohol Depend 34:231–236. 8033761 10.1016/0376-8716(94)90161-9

[adb12503-bib-0035] Nutt DJ , King LA , Phillips LD , Drugs ISC (2010) Drug harms in the UK: a multicriteria decision analysis. Lancet 376:1558–1565. 21036393 10.1016/S0140-6736(10)61462-6

[adb12503-bib-0036] Paterson LM , Flechais RS , Murphy A , Reed LJ , Abbott S , Boyapati V , Elliott R , Erritzoe D , Ersche KD , Faluyi Y , Faravelli L , Fernandez‐Egea E , Kalk NJ , Kuchibatla SS , McGonigle J , Metastasio A , Mick I , Nestor L , Orban C , Passetti F , Rabiner EA , Smith DG , Suckling J , Tait R , Taylor EM , Waldman AD , Robbins TW , Deakin JW , Nutt DJ , Lingford‐Hughes AR , Platform I (2015) The Imperial College Cambridge Manchester (ICCAM) platform study: an experimental medicine platform for evaluating new drugs for relapse prevention in addiction. Part A: Study description. J Psychopharmacol 9:943–960. 10.1177/0269881115596155 26246443

[adb12503-bib-0037] Power JD , Barnes KA , Snyder AZ , Schlaggar BL , Petersen SE (2012) Spurious but systematic correlations in functional connectivity MRI networks arise from subject motion. Neuroimage 59:2142–2154. 22019881 10.1016/j.neuroimage.2011.10.018 PMC3254728

[adb12503-bib-0038] Rubinov M , Sporns O (2010) Complex network measures of brain connectivity: uses and interpretations. Neuroimage 52:1059–1069. 19819337 10.1016/j.neuroimage.2009.10.003

[adb12503-bib-0039] Schmidt WK , Tam SW , Shotzberger GS , Smith DH , Clark R , Vernier VG (1985) Nalbuphine. Drug Alcohol Depend 14:339–362. 2986929 10.1016/0376-8716(85)90066-3

[adb12503-bib-0040] Spanagel R , Herz A , Shippenberg TS (1992) Opposing tonically active endogenous opioid systems modulate the mesolimbic dopaminergic pathway. Proc Natl Acad Sci U S A 89:2046–2050. 1347943 10.1073/pnas.89.6.2046 PMC48593

[adb12503-bib-0041] Sporns O , Chialvo DR , Kaiser M , Hilgetag CC (2004) Organization, development and function of complex brain networks. Trends Cogn Sci 8:418–425. 15350243 10.1016/j.tics.2004.07.008

[adb12503-bib-0042] Streeton C , Whelan G (2001) Naltrexone, a relapse prevention maintenance treatment of alcohol dependence: a meta‐analysis of randomized controlled trials. Alcohol Alcohol 36:544–552. 11704620 10.1093/alcalc/36.6.544

[adb12503-bib-0043] Sturgess JE , George TP , Kennedy JL , Heinz A , Muller DJ (2011) Pharmacogenetics of alcohol, nicotine and drug addiction treatments. Addict Biol 16:357–376. 21362114 10.1111/j.1369-1600.2010.00287.x

[adb12503-bib-0044] Tiffany ST , Friedman L , Greenfield SF , Hasin DS , Jackson R (2012) Beyond drug use: a systematic consideration of other outcomes in evaluations of treatments for substance use disorders. Addiction 107:709–718. 21981638 10.1111/j.1360-0443.2011.03581.x PMC3257402

[adb12503-bib-0045] Tzourio‐Mazoyer N , Landeau B , Papathanassiou D , Crivello F , Etard O , Delcroix N , Mazoyer B , Joliot M (2002) Automated anatomical labeling of activations in SPM using a macroscopic anatomical parcellation of the MNI MRI single‐subject brain. Neuroimage 15:273–289. 11771995 10.1006/nimg.2001.0978

[adb12503-bib-0046] Verdejo‐Garcia AJ , Perales JC , Perez‐Garcia M (2007) Cognitive impulsivity in cocaine and heroin polysubstance abusers. Addict Behav 32:950–966. 16876962 10.1016/j.addbeh.2006.06.032

[adb12503-bib-0047] Verdejo‐Garcia A , Perez‐Garcia M (2007) Profile of executive deficits in cocaine and heroin polysubstance users: common and differential effects on separate executive components. Psychopharmacology (Berl) 190:517–530. 17136401 10.1007/s00213-006-0632-8

[adb12503-bib-0048] Verebey K , Mule SJ (1975) Naltrexone pharmacology, pharmacokinetics, and metabolism — current status. Am J Drug Alcohol Abuse 2:357–363. 1227297 10.3109/00952997509005661

[adb12503-bib-0049] Volkow ND , Fowler JS (2000) Addiction, a disease of compulsion and drive: involvement of the orbitofrontal cortex. Cereb Cortex 10:318–325. 10731226 10.1093/cercor/10.3.318

[adb12503-bib-0050] Wang Z , Suh J , Li Z , Li Y , Franklin T , O'Brien C , Childress AR (2015) A hyper‐connected but less efficient small‐world network in the substance‐dependent brain. Drug Alcohol Depend 152:102–108. 25957794 10.1016/j.drugalcdep.2015.04.015 PMC4458212

[adb12503-bib-0051] Watts DJ , Strogatz SH (1998) Collective dynamics of 'small‐world' networks. Nature 393:440–442. 9623998 10.1038/30918

[adb12503-bib-0052] Weiland BJ , Sabbineni A , Calhoun VD , Welsh RC , Bryan AD , Jung RE , Mayer AR , Hutchison KE (2014) Reduced left executive control network functional connectivity is associated with alcohol use disorders. Alcohol Clin Exp Res 38:2445–2453. 25257293 10.1111/acer.12505 PMC4180110

[adb12503-bib-0053] Wise RA (1988) The neurobiology of craving — implications for the understanding and treatment of addiction. J Abnorm Psychol 97:118–132. 3290303 10.1037//0021-843x.97.2.118

[adb12503-bib-0054] Yuan K , Qin W , Liu JX , Guo QA , Dong MH , Sun JB , Zhang Y , Liu P , Wang W , Wang YR , Li QA , Yang WC , von Deneen KM , Gold MS , Liu YJ , Tian J (2010) Altered small‐world brain functional networks and duration of heroin use in male abstinent heroin‐dependent individuals. Neurosci Lett 477:37–42. 20417253 10.1016/j.neulet.2010.04.032

[adb12503-bib-0055] Zalesky A , Fornito A , Bullmore ET (2010) Network‐based statistic: identifying differences in brain networks. Neuroimage 53:1197–1207. 20600983 10.1016/j.neuroimage.2010.06.041

