# Supplementary material for: Naltrexone ameliorates functional network abnormalities in alcohol‐dependent individuals
Source: Addict Biol. 2017 Feb 28;23(1):425–36. doi: 10.1111/adb.12503 (PMC5811832; doi:10.1111/adb.12503)
Supplement: Supplementary file 1 — Table S1. Regions with reduced nodal efficiency in alcohol dependent (Alc) individuals compared to healthy volunteers (HV). Local efficiency for each node was calculated and compared between groups during placebo and naltrexone. Mean and standard error of the mean (s.e.m) are demonstrated and P‐values for both uncorrected and Bonferroni correction for multiple comparisons tests are displayed. Left, L; Right, R. Table S2. Regions demonstrating reduced functional connectivity in alcohol dependent subjects compared to healthy volunteers. Network based statistics revealed a significant cluster of reduced functional connectivity including 69 nodes and 373 edges. The number indicates the number of connections included in the significantly reduced cluster. Temporal and inferior frontal regions seemed to show the highest reduction in network connectivity. Left, L; Right, R. Table S3. Regions demonstrating reduced functional connectivity in alcohol dependent subjects compared to poly‐drug dependent subjects. The number indicates the number of connections included in the significantly reduced cluster. Frontal regions show the highest reduction in network connectivity. Left, L; Right, R. Table S4. Regions demonstrating reduced functional connectivity in alcohol dependent subjects compared to healthy volunteers during baseline session. The number indicates the number of connections included in the significantly reduced cluster. Left, L; Right, R. Table S5. Regions demonstrating reduced functional connectivity in alcohol dependent subjects compared to healthy volunteers during naltrexone. The number indicates the number of connections included in the significantly reduced cluster. Left, L; Right, R [file ADB-23-425-s001.docx]

Supplementary Materials

Methods

During the initial baseline assessment, written informed consent was obtained and eligibility was assessed with psychiatric interview. The Alcohol Use Disorders Identification Test (AUDIT)(Saunders et al., 1993) was administered alongside the ASSIST and measures of lifetime drug use for each substance. Alcohol and drug exposure was determined by a clinician based on the following criteria: for AD, a cumulative alcohol exposure measure was calculated in whole years using timeline follow-back(LC Sobell, 1992). Years of regular alcohol use was measured and one year was defined when meeting the following criteria for >6 months in a 12 month period in men more than 400g per week, or more than 3 binges of > 70g per week; and in women, more than 280g per week, or more than 3 binges of  > 56g per week(Organisation, 1999). One year of cocaine exposure was recorded if >6 months in a 12 month period involved >1 use per week of ≥1g. One year of heroin exposure was recorded if >6 months in a 12 month period involved >1 use per week of any amount. Participants additionally completed the Beck Depression Inventory -II(AT Beck, 1996) for depressive symptoms and the state-trait anxiety index (STAI)(Spielberger, 2010) for anxiety symptoms. Other cognitive, behavioural, physiological tests not reported here were additionally employed.

Data were collected from three centres in the UK: Imperial College (Imanova Limited), London; The Wolfson Brain Imaging Centre, University of Cambridge; Salford Royal NHS Foundation Trust, Manchester. All data were collected with 3T scanners with London and Cambridge using nominally identical 3T Siemens Tim Trio systems with a Siemens 32 channel head coil. Manchester used a 3T Philips Achieva with an 8 element SENSE head coil. Structural MRI data were acquired with high-resolution T1-weighted volumes using a magnetization-prepared rapid gradient echo (MPRAGE) sequence. A recent report(McGonigle et al., 2016) demonstrated no differences between centres for neuroimaging results during task performance, using the exact same data acquisition sequences and processing in healthy volunteers.

In London and Cambridge, functional imaging consisted of a multi-echo gradient echo echoplanar imaging (EPI) sequence (TR= 2000ms; TE= 13 and 31ms; flip angle= 80^o^; field of view= 225mm; image matrix= 64 x 64) with a 3.5 x 3.5mm in plane resolution and 3mm slice thickness. Phase encoding direction was anterior to posterior with an echo spacing of 0.52ms. For the 180 volumes, 36 abutting ascending oblique axial slices were collected. Generalized Autocalibrating Partially Parallel Acquisition (GRAPPA) imaging with an acceleration factor of 2 was employed. The first 3 volumes were discarded to allow for T1 saturation effects. In Manchester, identical EPI parameters were used but with 34 slices with an acceleration using Sensitivity Encoding (SENSE).

Results

The following were additionally included as covariates in this main analysis of local efficiency: site, gender, age, smoking status, depression score and anxiety score. There was no main effect of any of these potentially confounding variables on local efficiency. There were no interactions between drug and any of these factors. There was no significant interaction between site and group (p=0.139) or smoking status and group (p=0.629).The drug x group interaction remained significant when controlling for all covariates in the model (p=0.049). The main effect of drug however was not significant (p>0.05).

Additional analysis of group effects during placebo revealed a significant effect of group (F_(3,90)_=2.922, p=0.023) on local efficiency. Post hoc independent samples t-test comparing AD and poly-drug SD with HV showed elevations in local efficiency in both AD versus HV (t_(55)_=-2.197, p=0.032), and poly drug SD versus HV (t_(70)_=-2.427, p=0.018).

Supplementary Tables

|  | Alc (mean) | HV (mean) | Alc (s.e.m) | HV (s.e.m) | p-value | Bonferroni correction |
| --- | --- | --- | --- | --- | --- | --- |
| *Nodal Efficiency (Placebo)* | |  |  |  |  |  |
| Temporal_Mid_L | 0.30 | 0.57 | 0.08 | 0.05 | **0.005** | 0.479 |
| Rectus_L | 0.59 | 0.24 | 0.10 | 0.07 | **0.008** | 0.705 |
| Supp_Motor_Area_R | 0.79 | 0.60 | 0.05 | 0.06 | **0.013** | 1.209 |
| Frontal_Mid_L | 0.69 | 0.52 | 0.05 | 0.04 | **0.017** | 1.494 |
| Temporal_Mid_R | 0.35 | 0.57 | 0.08 | 0.04 | **0.018** | 1.609 |
| Rectus_R | 0.47 | 0.23 | 0.09 | 0.07 | **0.040** | 3.573 |
| Occipital_Mid_R | 0.86 | 0.76 | 0.02 | 0.04 | **0.043** | 3.863 |
| ParaHippocampal_L | 0.21 | 0.03 | 0.08 | 0.03 | **0.046** | 4.156 |
| Olfactory_R | 0.15 | 0.00 | 0.07 | 0.00 | **0.048** | 4.358 |
| Temporal_Pole_Sup_L | 0.33 | 0.14 | 0.09 | 0.04 | 0.052 | 4.650 |
| Amygdala_L | 0.15 | 0.02 | 0.07 | 0.02 | 0.074 | 6.662 |
| SupraMarginal_L | 0.40 | 0.62 | 0.10 | 0.06 | 0.075 | 6.718 |
| Olfactory_L | 0.17 | 0.03 | 0.08 | 0.02 | 0.089 | 8.023 |
| Frontal_Sup_Orb_L | 0.38 | 0.20 | 0.09 | 0.05 | 0.090 | 8.114 |
| *Nodal Efficiency (Naltrexone)* | |  |  |  |  |  |
| Cingulum_Mid_R | 0.74 | 0.60 | 0.04 | 0.05 | **0.017** | 1.574 |
| Temporal_Mid_L | 0.29 | 0.44 | 0.06 | 0.05 | 0.053 | 4.753 |
| Temporal_Pole_Sup_R | 0.13 | 0.28 | 0.05 | 0.06 | 0.054 | 4.880 |
| Temporal_Mid_R | 0.36 | 0.51 | 0.07 | 0.05 | 0.070 | 6.329 |
| Cingulum_Mid_L | 0.67 | 0.57 | 0.03 | 0.04 | 0.081 | 7.260 |
| Supp_Motor_Area_L | 0.79 | 0.64 | 0.06 | 0.06 | 0.081 | 7.278 |
| Temporal_Pole_Sup_L | 0.10 | 0.26 | 0.07 | 0.06 | 0.091 | 8.179 |
| Supp_Motor_Area_R | 0.77 | 0.63 | 0.06 | 0.05 | 0.097 | 8.773 |
| Olfactory_L | 0.00 | 0.07 | 0.00 | 0.04 | 0.102 | 9.165 |
| Temporal_Sup_L | 0.54 | 0.65 | 0.06 | 0.03 | 0.110 | 9.915 |
| Occipital_Inf_R | 0.56 | 0.75 | 0.11 | 0.05 | 0.118 | 10.632 |
| Angular_L | 0.14 | 0.28 | 0.06 | 0.06 | 0.133 | 11.999 |
| Precentral_R | 0.79 | 0.70 | 0.05 | 0.04 | 0.141 | 12.697 |
| Precuneus_L | 0.45 | 0.57 | 0.06 | 0.05 | 0.166 | 14.935 |

Supplementary Table 1. Regions with reduced nodal efficiency in alcohol dependent (Alc) individuals compared to healthy volunteers (HV). Local efficiency for each node was calculated and compared between groups during placebo and naltrexone. Mean and standard error of the mean (s.e.m) are demonstrated and p-values for both uncorrected and Bonferroni correction for multiple comparisons tests are displayed. Left, L; Right, R.

| Region | Connections | Region | Connections | Region | Connections |
| --- | --- | --- | --- | --- | --- |
| Temporal_Mid_L | 28 | Occipital_Sup_R | 12 | Frontal_Mid_Orb_L | 1 |
| Temporal_Inf_R | 26 | Cingulum_Mid_R | 11 | Frontal_Med_Orb_L | 1 |
| SupraMarginal_R | 25 | Occipital_Mid_R | 11 | Frontal_Med_Orb_R | 1 |
| Temporal_Sup_L | 25 | Rolandic_Oper_L | 10 | Hippocampus_L | 1 |
| Temporal_Mid_R | 24 | Cingulum_Ant_L | 10 | Calcarine_L | 1 |
| Frontal_Inf_Tri_L | 23 | Frontal_Inf_Oper_L | 9 | Angular_L | 1 |
| Temporal_Sup_R | 23 | Parietal_Inf_R | 9 | Putamen_R | 1 |
| Supp_Motor_Area_R | 21 | Angular_R | 9 | Pallidum_R | 1 |
| Heschl_L | 21 | Paracentral_Lobule_R | 9 | Temporal_Inf_L | 1 |
| Frontal_Mid_R | 19 | Frontal_Sup_R | 8 |  |  |
| Parietal_Inf_L | 19 | ParaHippocampal_R | 8 |  |  |
| SupraMarginal_L | 19 | Postcentral_R | 8 |  |  |
| Precuneus_R | 19 | Frontal_Sup_Medial_R | 7 |  |  |
| Precentral_R | 18 | Cuneus_R | 7 |  |  |
| Precentral_L | 17 | Lingual_L | 7 |  |  |
| Frontal_Inf_Oper_R | 17 | Insula_R | 6 |  |  |
| Frontal_Inf_Tri_R | 16 | Occipital_Mid_L | 6 |  |  |
| Cingulum_Ant_R | 15 | Paracentral_Lobule_L | 6 |  |  |
| Cingulum_Mid_L | 15 | Temporal_Pole_Mid_R | 6 |  |  |
| Fusiform_R | 15 | Frontal_Mid_L | 5 |  |  |
| Postcentral_L | 15 | Occipital_Inf_L | 5 |  |  |
| Frontal_Mid_Orb_R | 14 | Frontal_Sup_L | 4 |  |  |
| Rolandic_Oper_R | 14 | Cingulum_Post_R | 4 |  |  |
| Lingual_R | 14 | Occipital_Sup_L | 4 |  |  |
| Occipital_Inf_R | 14 | Cingulum_Post_L | 3 |  |  |
| Precuneus_L | 14 | Calcarine_R | 3 |  |  |
| Supp_Motor_Area_L | 13 | Temporal_Pole_Sup_R | 3 |  |  |
| Frontal_Sup_Medial_L | 13 | Frontal_Sup_Orb_R | 2 |  |  |
| Fusiform_L | 13 | Frontal_Inf_Orb_L | 2 |  |  |
| Cuneus_L | 12 | Insula_L | 2 |  |  |

Supplementary Table 2. Regions demonstrating reduced functional connectivity in alcohol dependent subjects compared to healthy volunteers. Network based statistics revealed a significant cluster of reduced functional connectivity including 69 nodes and 373 edges. The number indicates the number of connections included in the significantly reduced cluster. Temporal and inferior frontal regions seemed to show the highest reduction in network connectivity. Left, L; Right, R.

| Region | Connections | Region | Connections |
| --- | --- | --- | --- |
| ParaHippocampal_R | 13 | Precuneus_R | 3 |
| Frontal_Sup_Medial_L | 12 | Heschl_L | 3 |
| Frontal_Sup_Medial_R | 12 | Frontal_Sup_Orb_L | 2 |
| Frontal_Inf_Tri_R | 10 | Frontal_Mid_R | 2 |
| Frontal_Mid_Orb_L | 9 | Frontal_Inf_Oper_R | 2 |
| Frontal_Mid_Orb_R | 8 | Supp_Motor_Area_L | 2 |
| SupraMarginal_R | 8 | Frontal_Med_Orb_L | 2 |
| Precentral_R | 7 | Cingulum_Mid_R | 2 |
| Temporal_Sup_R | 7 | Calcarine_L | 2 |
| SupraMarginal_L | 6 | Lingual_L | 2 |
| Frontal_Inf_Tri_L | 5 | Occipital_Mid_L | 2 |
| ParaHippocampal_L | 5 | Occipital_Mid_R | 2 |
| Fusiform_L | 5 | Occipital_Inf_L | 2 |
| Angular_R | 5 | Occipital_Inf_R | 2 |
| Temporal_Sup_L | 5 | Parietal_Sup_L | 2 |
| Frontal_Sup_R | 4 | Temporal_Mid_L | 2 |
| Rolandic_Oper_R | 4 | Temporal_Pole_Mid_R | 2 |
| Cingulum_Ant_L | 4 | Precentral_L | 1 |
| Cingulum_Ant_R | 4 | Frontal_Mid_L | 1 |
| Cingulum_Mid_L | 4 | Rectus_R | 1 |
| Fusiform_R | 4 | Cuneus_R | 1 |
| Precuneus_L | 4 | Postcentral_L | 1 |
| Temporal_Inf_R | 4 | Parietal_Sup_R | 1 |
| Frontal_Inf_Orb_L | 3 | Parietal_Inf_R | 1 |
| Supp_Motor_Area_R | 3 | Angular_L | 1 |
| Frontal_Med_Orb_R | 3 | Paracentral_Lobule_L | 1 |
| Amygdala_L | 3 | Putamen_R | 1 |
| Calcarine_R | 3 | Temporal_Pole_Sup_L | 1 |
| Cuneus_L | 3 | Temporal_Pole_Sup_R | 1 |
| Lingual_R | 3 | Temporal_Mid_R | 1 |

Supplementary Table 3. Regions demonstrating reduced functional connectivity in alcohol dependent subjects compared to poly-drug dependent subjects. The number indicates the number of connections included in the significantly reduced cluster. Frontal regions show the highest reduction in network connectivity. Left, L; Right, R.

| Region | Connections | Region | Connections |
| --- | --- | --- | --- |
| Paracentral_Lobule_R | 19 | Precentral_L | 1 |
| Supp_Motor_Area_R | 9 | Frontal_Sup_R | 1 |
| Fusiform_L | 9 | Frontal_Mid_L | 1 |
| Amygdala_R | 5 | Frontal_Mid_R | 1 |
| Fusiform_R | 5 | Frontal_Inf_Oper_R | 1 |
| SupraMarginal_L | 5 | Frontal_Inf_Tri_R | 1 |
| Putamen_R | 4 | Rolandic_Oper_R | 1 |
| Temporal_Mid_L | 4 | Supp_Motor_Area_L | 1 |
| Occipital_Sup_L | 3 | Frontal_Sup_Medial_L | 1 |
| Occipital_Mid_L | 3 | Cingulum_Ant_L | 1 |
| Precentral_R | 2 | Cingulum_Ant_R | 1 |
| Frontal_Inf_Tri_L | 2 | Cingulum_Mid_L | 1 |
| Frontal_Sup_Medial_R | 2 | Cingulum_Mid_R | 1 |
| Amygdala_L | 2 | Cingulum_Post_R | 1 |
| Cuneus_R | 2 | Cuneus_L | 1 |
| Occipital_Sup_R | 2 | Occipital_Inf_L | 1 |
| Occipital_Mid_R | 2 | Occipital_Inf_R | 1 |
| SupraMarginal_R | 2 | Postcentral_L | 1 |
| Temporal_Sup_R | 2 | Postcentral_R | 1 |
| Temporal_Mid_R | 2 |  |  |

Supplementary Table 4. Regions demonstrating reduced functional connectivity in alcohol dependent subjects compared to healthy volunteers during baseline session. The number indicates the number of connections included in the significantly reduced cluster. Left, L; Right, R.

| Region | Connections | Region | Connections |
| --- | --- | --- | --- |
| Angular_R | 24 | Frontal_Inf_Tri_L | 4 |
| Frontal_Sup_Medial_R | 19 | Insula_L | 4 |
| Precuneus_R | 14 | Cuneus_L | 4 |
| Thalamus_L | 13 | Fusiform_L | 4 |
| Occipital_Sup_R | 12 | Parietal_Inf_L | 4 |
| SupraMarginal_R | 12 | Putamen_R | 4 |
| Cingulum_Mid_R | 11 | Rolandic_Oper_L | 3 |
| Frontal_Sup_Medial_L | 10 | Rolandic_Oper_R | 3 |
| Temporal_Sup_R | 10 | Supp_Motor_Area_L | 3 |
| Frontal_Inf_Tri_R | 9 | Occipital_Sup_L | 3 |
| Hippocampus_L | 9 | Fusiform_R | 3 |
| Occipital_Mid_R | 9 | Frontal_Sup_R | 2 |
| Parietal_Inf_R | 9 | Frontal_Sup_Orb_R | 2 |
| Cingulum_Ant_R | 8 | Frontal_Mid_Orb_R | 2 |
| Lingual_R | 8 | Frontal_Inf_Orb_R | 2 |
| Pallidum_R | 8 | Rectus_L | 2 |
| Temporal_Mid_R | 8 | Cingulum_Post_R | 2 |
| Cingulum_Mid_L | 7 | Hippocampus_R | 2 |
| Cingulum_Post_L | 7 | Cuneus_R | 2 |
| Pallidum_L | 7 | Lingual_L | 2 |
| Temporal_Mid_L | 7 | Angular_L | 2 |
| Precentral_R | 6 | Precuneus_L | 2 |
| Frontal_Med_Orb_R | 6 | Temporal_Pole_Sup_L | 2 |
| Insula_R | 6 | Frontal_Inf_Oper_L | 1 |
| ParaHippocampal_L | 6 | Frontal_Inf_Orb_L | 1 |
| Postcentral_R | 6 | Frontal_Med_Orb_L | 1 |
| SupraMarginal_L | 6 | Rectus_R | 1 |
| Thalamus_R | 6 | Amygdala_L | 1 |
| Temporal_Sup_L | 6 | Occipital_Inf_R | 1 |
| Frontal_Mid_R | 5 | Postcentral_L | 1 |
| Supp_Motor_Area_R | 5 | Paracentral_Lobule_R | 1 |
| Cingulum_Ant_L | 5 | Putamen_L | 1 |
| Temporal_Inf_R | 5 | Temporal_Pole_Mid_R | 1 |

Supplementary Table 5. Regions demonstrating reduced functional connectivity in alcohol dependent subjects compared to healthy volunteers during naltrexone. The number indicates the number of connections included in the significantly reduced cluster. Left, L; Right, R.

Supplementary Figure Legend

Supplementary Figure 1. Neural network local efficiency under naltrexone. Local efficiency was captured based on a whole brain ROI-to-ROI correlation coefficient matrix, binarized with a 5% density threshold and is plotted for baseline, naltrexone and placebo for alcohol dependent (Alc, unbroken line), poly-substance dependent (Poly, broken line) and healthy volunteers (HV, dotted line). There was no difference in local efficiency across sessions in HV or Polysubjects. There was a significant difference across sessions in the Alc group, in which local efficiency was significantly reduced by naltrexone.

Supplementary Figure 2. Network cluster of reduced functional connectivity in alcohol dependent (AD) subjects during baseline session. Network based statistics demonstrated a large network of reduced connectivity in AD compared to healthy subjects. Node size indicates number of connections with reduced functional connectivity. The largest nodes are annotated.
