# Supplementary figures and images for: Naltrexone ameliorates functional network abnormalities in alcohol‐dependent individuals
Source: Addict Biol. 2017 Feb 28;23(1):425–36. doi: 10.1111/adb.12503 (PMC5811832; doi:10.1111/adb.12503)

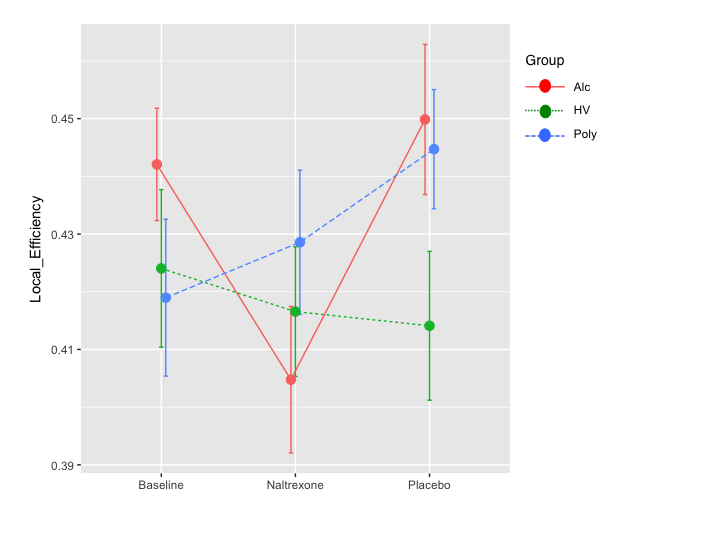

Supplement: Supplementary file 2 — Figure S1. Neural network local efficiency under naltrexone. Local efficiency was captured based on a whole brain ROI‐to‐ROI correlation coefficient matrix, binarized with a 5 percent density threshold and is plotted for baseline, naltrexone and placebo for alcohol dependent (Alc, unbroken line), poly‐substance dependent (Poly, broken line) and healthy volunteers (HV, dotted line). There was no difference in local efficiency across sessions in HV or Polysubjects. There was a significant difference across sessions in the Alc group, in which local efficiency was significantly reduced by naltrexone [file ADB-23-425-s002.tif]

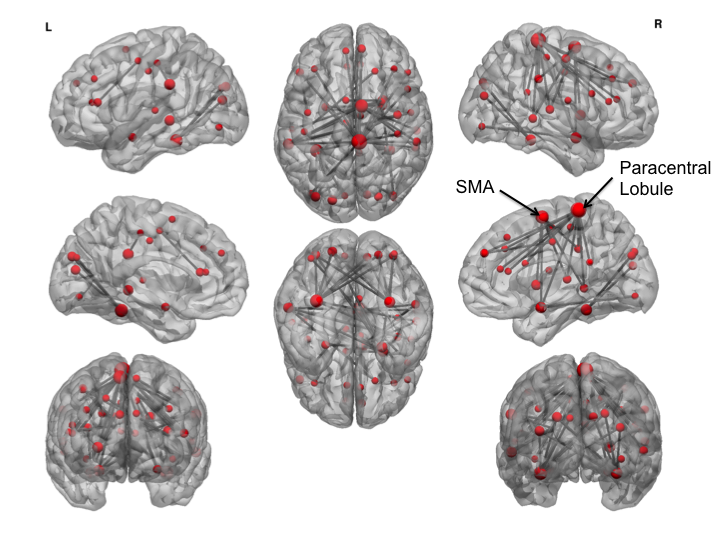

Supplement: Supplementary file 3 — Figure S2. Network cluster of reduced functional connectivity in alcohol dependent (AD) subjects during baseline session. Network based statistics demonstrated a large network of reduced connectivity in AD compared with healthy subjects. Node size indicates number of connections with reduced functional connectivity. The largest nodes are annotated [file ADB-23-425-s003.tif]
